# Effects of social health insurance on access and utilization of obstetric health services: results from HIV+ pregnant women in Kenya

**DOI:** 10.1186/s12889-020-8186-y

**Published:** 2020-01-20

**Authors:** Lawrence P. O. Were, Edwin Were, Richard Wamai, Joseph Hogan, Omar Galarraga

**Affiliations:** 1 Department of Health Sciences, Boston University’s College of Health and Rehabilitation Sciences: Sargent College, Boston, USA; 2 School of Public Health, Brown University, Providence, RI USA; 3 Department of Reproductive Health, Moi University & AMPATH-Kenya, Eldoret, Kenya; 4 Department of Cultures, Societies and Global Studies, Northeastern University, Boston, MA USA

**Keywords:** Healthcare utilization, HIV/AIDS, Health insurance, Institutional delivery, Skilled birth attendants, Universal health coverage

## Abstract

**Background:**

Reducing maternal morbidity and mortality remains a top global health agenda especially in high HIV/AIDS endemic locations where there is increased likelihood of mother to child transmission (MTCT) of HIV. Social health insurance (SHI) has emerged as a viable option to improve population access to health services, while improving outcomes for disenfranchised populations, particularly HIV+ women. However, the effect of SHI on healthcare access for HIV+ persons in limited resource settings is yet to undergo rigorous empirical evaluation. This study analyzes the effect of health insurance on obstetric healthcare access including institutional delivery and skilled birth attendants for HIV+ pregnant women in Kenya.

**Methods:**

We analyzed cross-sectional data from HIV+ pregnant women (ages 15–49 years) who had a delivery (full term, preterm, miscarriage) between 2008 and 2013 with their insurance enrollment status available in the electronic medical records database of a HIV healthcare system in Kenya. We estimated linear and logistic regression models and implemented matching and inverse probability weighting (IPW) to improve balance on observable individual characteristics. Additionally, we estimated heterogeneous effects stratified by HIV disease severity (CD4 < 350 as “Severe HIV disease”, and CD4 > 350 otherwise).

**Findings:**

Health Insurance enrollment is associated with improved obstetric health services utilization among HIV+ pregnant women in Kenya. Specifically, HIV+ pregnant women covered by NHIF have greater access to institutional delivery (12.5-percentage points difference) and skilled birth attendants (19-percentage points difference) compared to uninsured. Notably, the effect of NHIF on obstetric health service use is much greater for those who are sicker (CD4 < 350) – 20 percentage points difference.

**Conclusion:**

This study confirms conceptual and practical considerations around health insurance and healthcare access for HIV+ persons. Further, it helps to inform relevant policy development for health insurance and HIV financing and delivery in Kenya and in similar countries in sub-Saharan Africa in the universal health coverage (UHC) era.

## Background

Reducing maternal mortality, a top global agenda for the past five decades has been central to the Millennium Development Goals (MDGs) and the ensuing Sustainable Development Goals (SDGs) [1] [2, 3]. While global maternal deaths have been declining, the situation is compounded by conditions such as HIV/AIDS that increase the risk of maternal death [4] [5]. Moreover, if these women develop obstetric complications especially in low-resource settings with no safety-net, they face catastrophic expenditures that threaten and push their households into poverty [6]. This also increases the likelihood of mother to child transmission (MTCT) of HIV [7]. Therefore, public investment in maternal healthcare to improve health outcomes is critical and greatly needed [8].

One strategy being implemented to reach the global goals for women’s health is universal health coverage (UHC). UHC calls for access of all people to comprehensive health services at affordable costs and without financial hardship through protection against catastrophic health expenditures [9]. Sub-Saharan Africa (SSA) especially needs UHC given its high burden of HIV/AIDS and maternal mortality, and weak healthcare systems [9–11]. Numerous studies have shown that many HIV+ women have reproductive desires and are having children [12–15]. The result has been an estimated 1.4 million women living with HIV getting pregnant [16] with these women more likely to experience negative health and economic outcomes and overall low quality of life [17]. There is thus the need for prevention of MTCT of HIV, through skilled care during delivery [18]. This need is further amplified by the fact that global health funders have focused largely on HIV treatment programs leading to relative neglect of other reproductive health priorities including maternal health [19] [20].

In line with the UHC agenda, social health insurance (SHI) – membership in a health insurance scheme among all in the population [21] – has emerged as a viable option to improve population access to health services, while improving outcomes for disenfranchised populations, particularly HIV+ women [22]. In SSA, Kenya offers a good opportunity to study the congruence of SHI, reproductive health and HIV related health outcomes as Kenya has been flagged as having potential to lead Africa towards UHC [23] [24]. Notably, Kenya has the oldest SHI in SSA: the National Hospital Insurance Fund (NHIF) [25] [26] and the 4th largest global HIV epidemic that is feminized as prevalence among women is 6.3% compared to men at 5.5% [27] [28].

### The National Hospital Insurance Fund (NHIF)

An Act of Parliament established the NHIF in 1966 [26]. The 1966 Act was driven by the government’s initial commitment to providing “free” health services as part of its development strategy to alleviate poverty and improve the welfare of Kenyans [26]. NHIF was mandated to deduct a graduated premium from wages and salaries of formal sector employees and covered the contributor, spouse, and children under 18 irrespective of the type of ailment [25]. However, efforts to expand coverage to all were constrained by several factors including a worsening HIV/AIDS crisis, a strained healthcare budget, and declining economic growth that pushed labor into the informal sector and affected the number of formal employees paying into the fund [25]. In 1998, NHIF was transformed from a government department to an autonomous parastatal [29]. The reform legislation provided for the NHIF to expand population access to high quality and affordable healthcare, be self-financing, monitor its own collections, distribute benefits to providers, and to make loans from its reserves to hospitals for service improvement [29]. Since then, the NHIF has instituted reforms that include management of inpatient and outpatient schemes for government employees; introduction of a quality improvement system; launch of health subsidies for the poor; revision of monthly premiums and increase in provider reimbursement rates through capitation; and accreditation of public and private hospitals to expand the provider network [30] [31] [32]. Under NHIF legislation, enrollment is based on which segment of the economy one is employed in. All formal sector employees and civil servants (government employees including military personnel) are mandatory members and their employers are obligated to deduct their monthly premiums from a portion of their gross salary [31]. On the other hand, informal sector workers and the self-employed can join voluntarily paying a flat monthly premium [31]. While there are no co-payments or co-insurance, those not enrolled in NHIF depend on out-of-pocket OOP or tax subsidized care in government facilities where quality is questionable [32]. However, the historical or contemporary impact of NHIF on members’ utilization and access to healthcare for high-risk populations has not previously undergone rigorous empirical evaluation.

This paper thus analyzes the impact of NHIF on access to obstetric healthcare services including institutional delivery and skilled birth attendants for HIV+ pregnant women in Kenya.

## Methods

### Hypothesis and study setting

We hypothesize that NHIF improves access and utilization of institutional delivery services, and maternal health and hence influences the well-being of HIV+ pregnant women. The hypothesis is based on economic theory suggesting that people purchase health insurance not only to avoid risk of financial loss, but also as a mechanism for gaining access to healthcare that would otherwise be unaffordable [33] [34]. We use data from the Academic Model Providing Access to Healthcare (AMPATH). AMPATH is one of the largest and most comprehensive HIV/AIDS healthcare systems in SSA providing care to more than 150,000 HIV+ individuals in Western Kenya, tests approximately 80,000 pregnant women annually for HIV, and has robust electronic medical records [35] [36]. AMPATH has also been at the forefront of helping the Kenya Ministry of Health (MOH) formulate and implement healthcare policy initiatives and changes [37].

### Study design and variables

The main data for this analysis comes from cross-sectional records of medical encounters for HIV+ individuals within the AMPATH system between 2008 and 2013. The data is stored in the AMPATH Medical Records System (AMRS) - an electronic database of clinical encounters spanning more than 500 healthcare facilities in Western Kenya with extensive socio-demographic, economic, and biological variables [35]. The study population includes HIV+ pregnant women (ages 15–49 years) who get their HIV care at AMPATH clinics and have had a delivery (full term, preterm, miscarriage) with their NHIF enrollment status available in the dataset. The study analytic samples comprise of HIV+ pregnant women whose information on their outcomes between 2008 and 2013 is complete. The Institutional Delivery and Skilled Birth Attendant samples are generated by intersecting the observed outcomes and reported insurance status leading to a cross-sectional dataset of the most recent observed outcomes.

The independent variable is *NHIF enrollment* (Yes/No). The outcome or dependent variables are *institutional delivery* (birth at a hospital: Yes/No) and *skilled birth attendant* (SBA) -help by a nurse, doctor, or trained midwife at delivery: (Yes/No) - this is the WHO definition of SBA [38].

The dataset also includes the following covariates: age; number of children, access to electricity and piped water, education, Cluster of Differentiation antigen 4 (CD4) count, travel time to clinic, and clinic site (urban or rural). As detailed below, this high dimensional vector of covariates allows for appropriate regression adjustment and use of the observed characteristics of enrollment in NHIF to estimate the effect of insurance enrollment on outcomes based on matching methods.

### Statistical analysis

First, we conduct bivariate analysis to determine the nature and degree of the relationship between enrolling in NHIF and socio-economic and demographic variables. Next, we estimate the association between NHIF and obstetric healthcare access using unadjusted and adjusted linear and logistic models. Despite the outcomes being binary (0,1), we use both linear and logistic regression models given that unless the probabilities being modeled are extreme, then linear and logistic models fit equally well and the linear model in the econometric literature is favored for ease of interpretation [39] [40]. Additionally, if the probabilities are extreme i.e. closer to 0/1, then the logistic regression can suffer from complete separation, quasi-complete separation, and rare events bias especially in small samples [41].

To improve comparability as selection into insurance is not random, we implemented matching methods based on the conditional probability of enrolling in insurance given a set of observed covariates as defined by Rosenbaum and Rubin [42]. The matching estimation takes advantage of the covariates available in the dataset including their higher order terms (squares, cubes, and quadratics) and reduces bias due to differences in observed covariates thus balancing the covariates in the insured and uninsured groups [42]. After using the logit model in estimating the propensity scores and achieving balance of the propensity score between the insured and uninsured we estimate and report the ‘*average treatment effect on the treated*’ (ATT) i.e., the average response to treatment (insurance) for those pregnant women that enrolled in or were enrolled in health insurance. Specifically, we use three matching methods (*stratification, kernel, and radius*) to estimate ATT based on the propensity score [43]. The *Stratification* method consists of dividing the range of variation of the propensity score into intervals such that within each interval, treated and control units have on average the same propensity score [43]. Stratification method however discards observations in blocks where either treated or control units are absent [43]. In *Radius matching*, each treated unit is matched only with the control units whose propensity score falls into a predefined neighborhood of the propensity score of the treated unit – for this paper the radius is 0.01. While in *Kernel Matching*, those insured are matched with a weighted average of all uninsured with weights that are inversely proportional to the distance between the propensity scores of insured and uninsured [43]. Both Radius and Kernel matching help address the limitations of stratified matching.

Additionally, we implemented inverse probability weighting (IPW). IPW weights subjects by the inverse probability of treatment received thus creating a synthetic sample in which treatment assignment is independent of measured baseline covariates and allows one to obtain unbiased estimates of average treatment effects [44]. IPW was implemented given the potential for unequal probabilities of NHIF enrollment [45]. The results from the IPW estimates are our preferred results in line with the literature [44]. Given that, this study is an observational cross-sectional study with a single treatment variable, as discussed by Bender and Lange 2001, multiple test adjustments were not performed [46]. All statistical analyses were implemented in Stata 13 with the program *“pscore.ado”* used for matching estimation and all standard errors bootstrapped.

### Heterogeneous effects

Further, there is also the potential that the average effects estimated from the different models are heterogeneous for those with and without NHIF, and thus differ from the estimated average effects. The potential for impact heterogeneity is addressed by further stratifying the analysis based on HIV disease severity. HIV disease severity is defined using CD4 counts with CD4 < 350 as “Severe HIV disease,” and CD4 > 350 otherwise [47].

## Results

### Descriptive statistics and association between NHIF and access to care

The analytic samples comprise of 1247 women (7% insured) in the institutional delivery sample; and 1235 women (6% insured) in the SBA sample. Table 1 shows that the mean age at enrollment in the study sample is 29 years. The ages when first pregnant at AMPATH, and age when pregnancy is first identified at AMPATH, are 4 and 8 months respectively after enrollment. Worth noting too, is that the uninsured HIV+ women have considerable loss to follow-up during pregnancy compared to the NHIF enrollees.

**Table 1:** Demographic and Socio-Economic Characteristics of HIV+ Pregnant Women by Health Insurance Status within AMPATH Electronic Medical Records

Mean Values	Study Sample		Institutional Delivery Sample		Skilled Birth Attendant Sample	
	NHIF	Uninsured	NHIF	Uninsured	NHIF	Uninsured
Outcome at Baseline (%)			86.52 (34.35)	68.77 (46.37)	94.12 (23.70)	73.09 (44.37)
Current Age	31.26 (0.08)	31.83 (0.02)	30.75 (0.53)	30.97 (0.16)	31.97 (0.55)	31.00 (0.16)
Age at Enrollment	29.51 (5.61)	29.03 (5.86)	26.91 (4.47)	27.42 (5.02)	27.41 (4.24)	27.43 (5.04)
Age First Pregnant @ AMPATH	30.43 (5.92)	29.80 (6.15)	27.53 (4.74)	28.05 (5.32)	28.34 (4.76)	28.14 (5.39)
# of Children	2.25 (1.64)	3.01 (1.86)	2.09 (1.43)	2.74 (1.68)	2.18 (1.46)	2.85 (1.73)
Piped Water (%)	39.29 (47.35)	12.84 (35.89)	35.96 (48.26)	12.68 (33.31)	25.00 (43.62)	10.70 (30.94)
Electricity (%)	36.91 (46.88)	10.54 (33.72)	38.20 (48.86)	11.23 (31.58)	30.90 (46.54)	10.10 (30.16)
Ever Attended School (%)	97.82 (16.29)	92.17 (0.35)	98.87 (10.6)	99.30 (8.29)	94.10 (23.7)	93.15 (25.28)
Previous # of Pregnancies	2.84 (1.72)	3.47 (1.96)	2.79 (1.29)	3.37 (1.76)	2.75 (1.23)	3.45 (1.80)
CD4 @ Enrollment	338.24 (247.14)	359.24 (307.48)	410.40 (255.90)	396.49 (327.03)	381.72 (307.80)	349.47 (328.14)
Lost to Follow-up During Pregnancy (%)	1.71 (16.67)	1.00 (15.06)	5.62 (23.16)	1.55 (12.38)	0.00 (0)	1.50 (11.99)
Travel Time (Hours)	1.97 (0.99)	2.05 (0.98)	1.89 (0.94)	2.00 (0.95)	1.96 (1.00)	2.011 (0.95)
Urban Clinic (%)	70.06 (47.59)	44.80 (50.0)	69.70 (46.23)	42.06 (49.39)	69.12 (46.54)	37.53 (48.44)
Observations N	1997 (15.86%)	10,596 (84.14%)	89 (7.14%)	1158 (92.86%)	68 (5.51%)	1167 (94.49%)
Overall N	12,593		1247		1235	

Notes: The table shows the characteristics of HIV+ pregnant women in the AMPATH Medical Records System (AMRS) by their Health Insurance Status. The Study sample comprises of HIV+ pregnant women with information on their outcomes between 2008 and 2013 complete. The Institutional Delivery and Skilled Birth Attendant samples are generated by intersecting the observed outcomes and reported insurance status leading to a cross-sectional dataset of the most recent observed outcomes. In parentheses/brackets are the standard errors except for the “observations” row at the bottom of the table, which are percentages

On the socio-economic front, HIV+ pregnant women enrolled in NHIF are more likely to have piped water and electricity at home, but no differences in education compared to the uninsured. Further NHIF enrollees seem to have shorter travel times and are more likely to receive their HIV care in urban settings.

The bivariate analysis results (Table 2) show that in both study samples, there exist significant differences in the mean values for the covariates among those enrolled in NHIF and those not enrolled. These differences are however not significant for the variables: age first pregnant at AMPATH, CD4 at enrollment and site of HIV clinic. As such, CD4 at enrollment is used in heterogeneous analysis.

**Table 2:** Bivariate Analysis of Association between Outcomes and Explanatory Variables of HIV+ Pregnant Women within AMPATH Electronic Medical Records

Mean Values	Institutional Delivery Sample			Skilled Birth Attendant Sample		
	Yes	No	*P* Values	Yes	No	P Values
Current Age	30.80 (0.18)	31.35 (0.30)	0.11	30.90 (0.18)	31.49 (0.32)	0.10
Age at Enrollment	27.19 (0.16)	27.87 (0.27)	0.03**	27.24 (0.16)	27.96 (0.30)	0.03**
Age First Pregnant @ AMPATH	27.87 (0.18)	28.37 (0.29)	0.12	28.02 (0.18)	28.50 (0.31)	0.17
# of Children	2.56 (0.05)	3.03 (0.09)	0.00***	2.66 (0.05)	3.25 (0.10)	0.00***
Piped Water (%)	0.16 (0.01)	0.11 (0.02)	0.02**	0.13 (0.01)	0.06 (0.01)	0.00***
Electricity (%)	0.16 (0.01)	0.06 (0.01)	0.00***	0.14 (0.01)	0.02 (0.01)	0.00***
Ever Attended School (%)	0.99 (0.00)	0.99 (0.01)	0.34	0.94 (0.01)	0.90 (0.02)	0.01**
Previous # of Pregnancies	3.21 (0.06)	3.6 (0.09)	0.00***	3.28 (0.06)	3.77 (0.11)	0.00***
CD4 @ Enrollment	388.28 (9.45)	419.02 (21.00)	0.12	346.35 (9.43)	365.35 (23.81)	0.37
Lost to Follow-up During Pregnancy (%)	0.01 (0.00)	0.03 (0.01)	0.06*	0.01 (0.00)	0.03 (0.01)	0.04**
Travel Time (Hours)	1.96 (0.03)	2.08 (0.05)	0.04**	1.97 (0.03)	2.11 (0.05)	0.02**
Urban Clinic (%)	0.45 (0.02)	0.43 (0.03)	0.60	0.39 (0.02)	0.40 (0.03)	0.88
Observations N	874 (70.09%)	373 (29.91%)		917 (74.25%)	318 (25.75%)	
Overall N	1247			1235		

Notes: The table shows the results of bivariate analysis of the association between outcomes and explanatory variables of HIV+ Pregnant Women within AMPATH Medical Records System (AMRS). The *p*-values are based on a two-sample t-test with equal variance. In parentheses/brackets are standard errors except for the “observations” row at the bottom of the table, which are percentages. Significance levels: ***p < 0.01, **p < 0.05, *p < 0.1

Table 4 reports the linear and logistic regression models showing the association between NHIF and institutional delivery and access to SBA. The unadjusted linear and logistic models show that NHIF enrollees are 17 percentage points (3 times) more likely to deliver at an institution and 21 percentage points (6 times) more likely to have the services of a SBA at delivery. Both the adjusted linear and logistic models show attenuated but significant effects of having insurance on the two outcomes. This attenuation in effects might be indicative of confounding and the effect of selective enrollment into NHIF cannot be ruled out. Thus these potential limitations and biases are addressed through matching and IPW.

## Main results

The main results are based on the propensity score estimation where Table 3 shows that there are not any statistically significant differences between the insured and uninsured in both matched analytic samples. The effect estimates from the matching methods are presented in Table 5 where results of different matching specifications including stratified, radius, and kernel matching are shown for the matched samples only. The average effect estimates from inverse probability weighting by logistic regression are also reported. Specifically, HIV+ pregnant women with insurance are more likely to deliver at an institution. The effect estimates range from 10.2 percentage points for stratified matching to 15.5 percentage points for radius matching and 11.3 percentage points for kernel matching. The IPW estimate of 12.5 percentage points (2 times the odds) is almost an average of the three matching models. All the estimates are statistically significant at the 1 or 5 percent level.

**Table 3:** Test of Balance of the Mean Propensity Score by Blocks

Panel A: Institutional Delivery Sample			
Block	Insured	Uninsured	P-Value
Block 1	0.033	0.026	0.0395
Block 2	0.066	0.062	0.1060
Block 3	0.090	0.085	0.0305
Block 4	0.153	0.139	0.0208
Block 5	0.280	0.269	0.4175
Block 6	0.457	0.480	0.6198
*N* = 1137	89	1048	
Panel B: Skilled Birth Attendant Sample			
Block	Insured	Uninsured	P-Value
Block 1	0.028	0.025	0.3297
Block 2	0.073	0.070	0.4539
Block 3	0.135	0.138	0.5857
Block 4	0.256	0.250	0.7342
Block 5	0.462	0.492	0.4877
*N* = 1017	68	949	

Note: The above table reports the test of balance of the mean propensity score between the insured and uninsured by blocks. The results are from implementing *“pscore.ado”* program in Stata. The *p*-values are based on a two-sample *t*-test with equal variance. The *pscore* command fits a logit (probit is the default) model with a starting specification of linear terms without interactions or higher order terms. If balance in not achieved in a block, the sample in the block is split into equally spaced intervals, with higher order terms and interactions included, and the average propensity score of the treated and controls is re-tested until balance is achieved

For access to skilled birth attendants, the effect estimates of the impact of NHIF are 14.3 percentage points for stratified matching, 20.5 percentage points for radius matching, and 16.8 percentage points for kernel matching. The IPW effect estimates (OR = 5.17) are 1.6 percentage points higher than the average effect estimates from the matching models. All the estimates are statistically significant at the 1% level. For both outcomes, the matching based effect estimates are within the same range as the linear and logistic models presented in Table 4. This consistency in the magnitude of the effect estimates suggests robustness of the results to different specifications.

**Table 4:** Linear and Logistic Regression Estimates of the Effect of NHIF

	Institutional Delivery				Skilled Birth Attendant			
	1 Linear Unadjusted	2 Linear Adjusted	3 Logistic Unadjusted [Odds Ratio]	4 Logistic Adjusted [Odds Ratio]	5 Linear Unadjusted	6 Linear Adjusted	7 Logistic Unadjusted [Odds Ratio]	8 Logistic Adjusted [Odds Ratio]
NHIF	0.177*** (0.039)	0.118*** (0.05)	2.91*** (0.921)	2.34*** (0.832)	0.21*** (0.03)	0.161*** (0.037)	5.89*** (3.06)	5.08*** (2.80)
95% CI	0.10–0.25	0.04–0.20	1.56–5.41	1.16–4.70	0.15–0.27	0.09–0.23	2.13–16.3	1.73–14.97
N	1247	1247	1247	1247	1235	1235	1235	1233
Constant	0.688*** (0.014)	−1.06 (4.163)	2.21 (0.921)	0.001 (0.011)	0.731* (0.013)	0.907 (4.074)	2.72 (0.179)	0.51 (11.17)
R Squared	0.01	0.08	0.009	0.07	0.012	0.084	0.014	0.083

### Heterogeneous effects by HIV disease severity

As discussed above, there is the potential for heterogeneity in the average effects estimated in this study. HIV disease severity is used to test for heterogeneity, with the results shown in Table 6.

The heterogeneous estimates show that those with severe HIV disease, across the different matching methods given their insurance status, utilize more care compared to those of higher CD4 count (CD4 > 350). For example, the IPW estimates show a 20 percentage-points difference (OR = 4.09) for those enrolled in NHIF compared to those not insured with CD4 < 350. For access to SBA, there are similar differential access to care based on HIV disease state for those mothers with insurance compared to those not insured. These estimates are statistically significant at the 1% level.

## Discussion

In this study looking at the influence of enrolling in the NHIF on utilization of obstetric services among HIV + pregnant women in western Kenya, we find that insurance is consistently associated with higher service utilization across different model specifications. Specifically, Table 1 shows that those with insurance are different from those HIV+ pregnant women without insurance in terms of their sociodemographic characteristics - a difference that could be indicative of selective enrollment into NHIF. With SHI’s goal being to ensure equitable access to care, these differences in demographics need to be addressed if more Kenyans are to benefit from NHIF. The fact that the Kenyan government’s policies such as Vision 2030 are designed to help bridge this divide is encouraging.

Despite the differences identified in Table 1, the heterogeneous estimates in Tables 5 indicate that even with selection into NHIF, the sicker HIV+ mothers have greater access and utilization of institutional delivery and skilled birth attendants compared to those of higher CD4 levels given their enrollment in NHIF. This may not be optimal for insurance design but a positive for healthcare policy. Moreover, it seems that those with lower CD4 are using more services because of higher need and the enabling effect of insurance. We may thus infer that NHIF seems to have positive effects on the sickest and disenfranchised HIV+ pregnant mothers. Additionally, NHIF appears to work both at a practical and conceptual level and is therefore important for policy makers interested in UHC and SHI as a healthcare financing mechanism. However, it is hard to attribute this to any particular policy strategy in Kenya as NHIF is just undergoing reforms, and AMPATH in the period covered in this study had not instituted any social health insurance program or healthcare financing policy. Perhaps with the ongoing reforms, NHIF stands to provide greater benefits.

**Table 5:** Effect Estimates of the Impact of NHIF based on Matching Methods

	Institutional Delivery				Skilled Birth Attendant			
	1 Stratified	2 Radius	3 Kernel	4 IPW [OR]	5 Stratified	6 Radius	7 Kernel	8 IPW [OR]
Coefficients (ATT)	0.102**	0.155***	0.113**	2.14**	0.143***	0.205***	0.168***	5.17***
Std. Errors	0.042	0.040	0.046	0.82	0.034	0.034	0.034	3.19
T/Z- Statistic	2.416	3.835	2.437	1.99	4.221	6.063	4.984	2.67
N								
Analytic Sample	1247	1247	1247	852	1235	1235	1235	744
Treatment	89	87	89		67	64	68	
Control	1017	1002	1017		928	919	927	

**Notes**: In the Table 4 above, models 1 & 5 are unadjusted linear models and 3 & 7 are unadjusted logistic models. Models 2 & 6 are linear models with controls and 4 & 8 are logistic with controls. The vector of controls include: age, household characteristics, education, pregnancy history, HIV care, travel time to clinics, and clinic sites (urban or rural). Reported for models 3, 4, 7 & 8 are Odds Ratios [OR]. In parentheses are robust standard errors. Significance levels: ***p < 0.01, **p < 0.05, *p < 0.1. In Table 5**,** model 1 & 5 is Stratified matching; model 2 & 6 is Radius matching (radius = 0.01); model 3 & 7 is Kernel matching; and model 4 & 8 is Inverse Probability Weighting (IPW) by logistic regression and results are Odds Ratios [OR]. The standard errors are bootstrapped. Significance levels: ***p < 0.01, **p < 0.05, *p < 0.1. The results presented in the Table 5 above are for the matched samples only

**Table 6:** Heterogeneous Effect Estimates – HIV Disease Severity

Panel A: Institutional Delivery								
	HIV DISEASE							
	CD4 ≤ 350				CD4 > 350			
	1 Stratified	2 Radius	3 Kernel	4 IPW [OR]	5 Stratified	6 Radius	7 Kernel	8 IPW [OR]
Coefficients (ATT)	0.124**	0.18***	0.142**	4.09**	0.094	0.126	0.099	2.54*
Stand Errors	0.053	0.06	0.058	2.71	0.067	0.065	0.068	1.22
T/Z- Statistic	2.326	2.977	2.437	2.12	1.414	1.919	1.447	1.94
N								
Analytic Sample	640	640	640	640	607	607	607	607
Treatment	40	40	40		49	42	49	
Control	523	505	523		384	373	384	
Panel B: Skilled Birth Attendant								
	**CD4 ≤ 350**				**CD4 > 350**			
Coefficients (ATT)	0.116***	0.192***	0.154***	11.82**	0.177***	0.215***	0.187***	8.12**
Stand Errors	0.043	0.052	0.046	9.24	0.056	0.057	0.062	7.00
T/Z- Statistic	2.685	3.725	3.87	3.16	3.191	3.809	3.004	2.43
N								
Analytic Sample	705	705	705	705	530	530	530	530
Treatment	36	34	36		31	29	32	
Control	525	521	525		376	365	375	

Notes: In the above table model 1 & 5 is Stratified matching; model 2 & 6 is Radius matching (radius = 0.01); model 3 & 7 is Kernel matching; and model 4 & 8 is Inverse Probability Weighting (IPW) by logistic regression. The IPW results are Odds Ratios [OR] while the matching results are percentage points differences. The standard errors are bootstrapped. Significance levels: ***p < 0.01, **p < 0.05, *p < 0.1. The results presented in the table above are for the matched samples only

These findings are also consistent with the literature on multiple levels. First, given that eight months after enrollment, a positive pregnancy test result is identified, is an indicator that on average most mothers conceived and delivered after knowing their HIV+ status. This bolsters the literature that shows that women still have reproductive desires after knowing their HIV status [12–15].

Second, while this is the first study to look at the relationship between insurance and access to obstetric services for HIV+ pregnant women in a developing country context, and SSA in particular, it supports the findings from developed countries that show that insurance is generally associated with better health outcomes for HIV+ individuals [48] [49]. However, even the US studies did not focus on access to obstetric services for HIV+ pregnant women.

Third, the findings of the heterogeneous analysis fit within the frameworks that inform expansion of SHI. This supports the push by social scientists and policy makers for SHI as a viable option to improve access to RH services, while improving health outcomes for disenfranchised populations, particularly HIV+ women [17] [22]. Ultimately, any expansion of SHI to include HIV related care and treatment does raise the question of sustainability of health insurance. Beyond expanding the coverage pool to include as many as possible in the general population, sustainable SHI expansion would need to be buttressed by tax mechanisms such as general and progressive taxation [50].

However, these findings may be limited given the nature of and enrollment in NHIF, and the source of data. This is possible given non-random selection into NHIF and the fact that the AMPATH data is captured from clinical encounters and is not designed for evaluation of social programs. The study is also based on cross-sectional observational data that are limited in causal estimation compared to experimental study designs. Nonetheless, given the extensive data and covariates available, the study tries to mimic experimental designs using matching methods in the estimation of the relationship between NHIF and obstetric healthcare access.

As SHI programs become more widely utilized in LMIC, SHI may be a valid and cost effective means to ensure access to RH services for HIV+ pregnant women. These findings should therefore be informative for countries with similar or comparable programs. Additionally, the HIV pandemic in Africa is highly feminized, and pediatric HIV is unacceptably high, thus our findings should be reflective of most SSA countries where HIV is highly generalized just as in Kenya. Finally, the AMPATH HIV treatment and prevention model is a well-developed system that may limit the comparability of our findings to other less developed systems and settings; however, the results may provide an upper-bound estimate in terms of the RH and insurance impact possibilities given a relatively well funded and well-managed HIV/AIDS care and PMTCT program.

## Conclusion

Ultimately, this study shows that SHI improves utilization of obstetric services among HIV + pregnant women in Kenya. Other than answering the question on the impact of NHIF, this study is also policy relevant and timely. In Kenya, the ongoing reforms focus on NHIF as the primary vehicle for health care financing. Our findings show that despite reforms taking place with a limited evidence base, NHIF seems to be having an effect. This effect is taking place within the purview of NHIF’s original mission and design – coverage of hospitalizations and institutional delivery. With declining funding for HIV/AIDS programs and the push by donors for increased country-level financing of HIV care, these findings show that it is possible to forge synergies essential for effective and sustainable HIV/AIDS policy, financing and programming. These findings thus help inform the UHC and HIV policy discussions both in Kenya and in similar countries around the global South.

## Data Availability

The raw data analyzed for this study were obtained from AMPATH. The data is available from the corresponding author upon reasonable request, and after consultation and approval by the Institutional Research and Ethics Committee (IREC) at Moi University/Moi Teaching and Referral hospital in Eldoret, Kenya.

## Abbreviations

AIDS: Acquired Immune Deficiency Syndrome
AMPATH: Academic Model Providing Access to Healthcare
CD4: Cluster of Differentiation antigen 4
HIV: Human Immunodeficiency Virus
IPW: Inverse Probability Weighting
KDHS: Kenya Demographic and Health Survey
MDG: Millennium Development Goals
MMR: Maternal Mortality Rate
MOH: Ministry of Health
NGO: Non-Governmental Organizations
NHIF: National Health Insurance Fund
OOP: Out-of-Pocket
PMTCT: Prevention of Mother to Child Transmission
SBA: Skilled Birth Attendants
SDG: Sustainable Development Goals
SHI: Social Health Insurance
SPA: Service Provision Assessment
SSA: Sub-Saharan Africa
UHC: Universal Health Coverage
WHO: World Health Organization
